# Tropomyosin is no accurate marker allergen for diagnosis of shrimp allergy in Central Europe

**DOI:** 10.1111/all.15290

**Published:** 2022-04-01

**Authors:** João Grilo, Ute Vollmann, Martina Aumayr, Gunter J. Sturm, Barbara Bohle

**Affiliations:** ^1^ Department of Pathophysiology and Allergy Research Center for Pathophysiology, Infectiology and Immunology Medical University of Vienna Vienna Austria; ^2^ MacroArray Diagnostics Vienna Austria; ^3^ Allergy Outpatient Clinic Reumannplatz Vienna Austria; ^4^ Department of Dermatology and Venereology Medical University of Graz Graz Austria

## Funding information

This study was supported by the Austrian Science Fund (FWF), projects W1248, P32953, and I4437, the Austrian Jubiläumsfond, project ÖNB17947, and by the Danube Allergy Research Cluster, Country of Lower Austria, and Medical University of Vienna, Austria.

## CONFLICT OF INTEREST

GJS reports grants and personal fees from ALK Abelló and personal fees from Novartis, Bencard, and Allergopharma, during the conduct of the study. BB reports grants from Austrian Science Funds, Austrian Jubiläumsfonds, and Medical University of Vienna, during the conduct of the study and personal fees from AllergenOnline outside the submitted work. MA is employed by MacroArray Diagnostics, Vienna, Austria. The other authors have nothing to declare.

**FIGURE 1:**
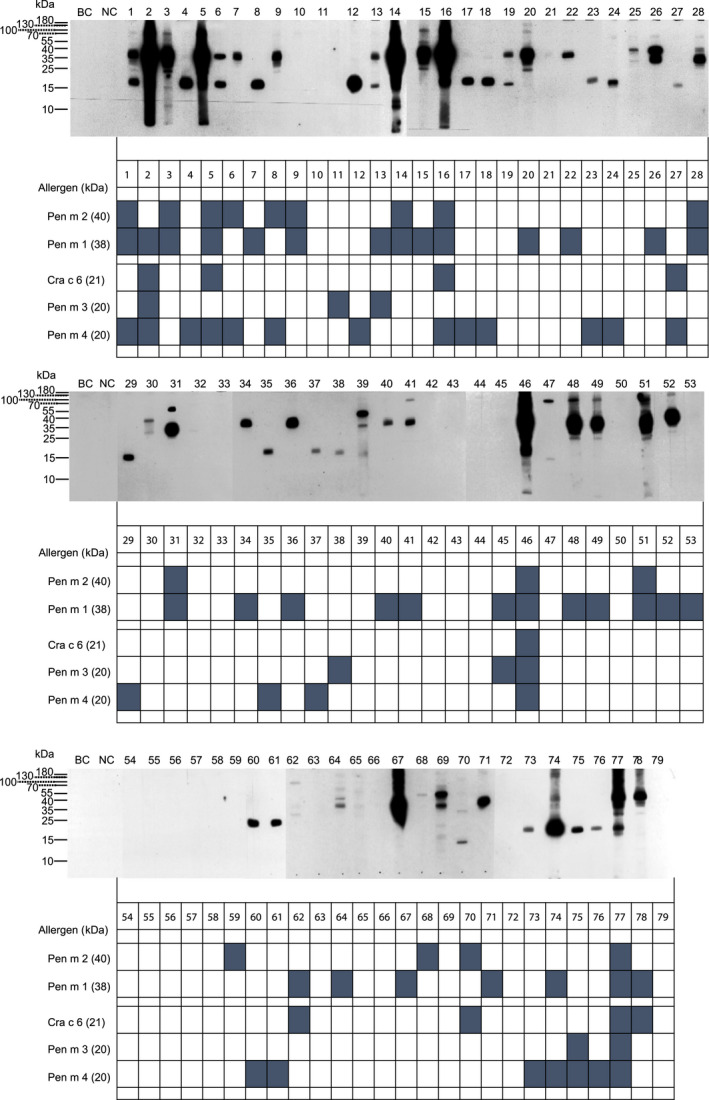
IgE‐profiling of 79 shrimp‐allergic individuals. IgE reactivity to *Litopenaeus vannamei* extract in immunoblot is shown in the upper panel. IgE reactivity to chip‐spotted allergens is represented as dark boxes in the lower panel; BC, buffer control, NC, non‐allergic control; kDa, kilo Dalton; detailed methodological information is provided in the online supplement

To the Editor,

*Crustacea* shrimp is a frequent cause of food allergy with symptoms ranging from mild oral reactions to severe anaphylaxis. Shrimp contains the single major allergen tropomyosin (TM), which is also present in several invertebrates, for example, house dust mites (HDM). In fact, shrimp allergy has initially been considered to result from IgE‐cross‐reactivity following sensitization to Der p 10, the TM from *Dermatophagoides pteronissimus*. Because TM‐specific IgE was found to be a good predictor of shrimp allergy in HDM‐allergic individuals,^1^ singleplex assays with shrimp TM found their way into serological routine diagnosis. However, evidence accumulated that additional allergens cause shrimp allergy and cross‐reactivity with HDM, such as arginine kinase (AK), myosin light chain (MLC), sarcoplasmic calcium‐binding protein (SCBP), hemocyanin (HC), triose‐phosphatase‐isomerase (TPI), and troponin C (TC).^2^ Here, samples from 79 individuals who experienced allergic reactions to shrimps and displayed shrimp‐specific IgE in ImmunoCAP (Table S1) were tested after approval by the ethics committee of the Medical University of Vienna (EK 1344/2018) and informed consent for IgE reactivity to recombinant allergens from *Penaeus monodon* and *Crangon crangon* in the newly developed Allergy Explorer‐ALEX 2^®^ and by immunoblotting to proteins extracted from *Litopenaeus vannamei*, a species frequently consumed in Austria, for example, as “cocktail shrimps” (for details see Appendix S1). The macroarray revealed that 42% of the patients displayed IgE to Pen m 1 (TM, 19% exclusively), 20% to Pen m 2 (AK, 3.7% exclusively), 10% to Pen m 3 (MLC, 2.5% exclusively), and 11% to Cra c 6 (TC), and matched IgE‐profiles determined by immunoblotting (Figure 1 and Table S2). Thus, TM represented no major allergen in this cohort. Notably, 30% of the patients recognized Pen m 4 and 16% showed exclusive IgE reactivity to this SCBP with confirmed clinical relevance.^3^ Therefore, we consider Pen m 4 as important as Pen m 1 for the diagnosis of shrimp allergy in Central Europe. This conclusion is in line with previous studies.^3^, ^4^, ^5^

In accordance with a strong association of shrimp and HDM allergy found by others,^6^ 75% of our patients were sensitized to HDM among who 54% reacted with Pen m 1, 27% with Pen m 2, 17% with Pen m 4, 14% with Cra c 6, and 10% with Pen m 3 (Table S2). Thus, Pen m 1 was the major allergen but no sole diagnostic marker of shrimp allergy as previously reported for Italian HDM‐allergic patients.^6^ We found no correlation of IgE levels specific for extracts from shrimp and HDM (*R* = .238, *p* = .072) and for HDM and Pen m 1 (*R* = .149, *p* = .216). Nevertheless, IgE levels for Pen m 1 and Der p 10 (*R* = .985, *p* < .001) and Pen m 2 and Der p 20 (*R* = .771, *p* < .001) correlated significantly. To confirm that additional allergens are required for an accurate serological diagnosis, we calculated the diagnostic sensitivity of Pen m 1 in combination with the other allergens (Table 1). All recombinant allergens together achieved a sensitivity of 68%. We expect that the inclusion of hemocyanin and ubiquitin might enhance this value as 10% and 9% of the patients recognized the corresponding proteins of 70 and 5–7 kDa, respectively, in the immunoblot (Figure 1). Hemocyanin was proposed to be relevant in the cross‐reactivity of shrimps and mites and is mainly contained in the cephalothorax, but may be found in the abdominal muscle. As we employed shrimps without cephalothorax for extract preparation, our results underestimate the frequency of hemocyanin recognition.

**TABLE 1:** IgE recognition of recombinant shrimp allergens of 59 HDM‐sensitized patients (%)

Pen m 1	54.2
Pen m 1 + Cra c 6	55.9
Pen m 1 + Pen m 3	57.6
Pen m 1 + Pen m 4	59.3
Pen m 1 + Pen m 3 + Pen m 4	59.3
Pen m 1 + Pen m 3 + Cra c 6	59.3
Pen m 1 + Pen m 4 + Cra c 6	61.0
Pen m 1 + Pen m 2	62.7
Pen m 1 + Pen m 2 + Cra c 6	62.7
Pen m 1 + Pen m 2 + Pen m 4	64.4
Pen m 1 + Pen m 2 + Pen m 4 + Cra c 6	64.4
Pen m 1 + Pen m 3 + Pen m 4 + Cra c 6	64.4
Pen m 1 + Pen m 2 + Pen m 3	66.1
Pen m 1 + Pen m 2 + Pen m 3 + Cra c 6	66.1
Pen m 1 + Pen m 2 + Pen m 3 + Pen m 4	67.8
Pen m 1 + Pen m 2 + Pen m 3 + Pen m 4 + Cra c 6	67.8

The molecular diagnosis approaches combined here further confirm how variate the IgE recognition of shrimp allergens can be, independently of whether patients are sensitized to HDM or not. This study shall raise awareness that TM is no accurate sole marker allergen for the serological diagnosis of shrimp allergy in HDM‐sensitized patients.

## Supporting information

App S1
