# Supplementary material for: Tropomyosin is no accurate marker allergen for diagnosis of shrimp allergy in Central Europe
Source: Allergy. 2022 Apr 1;77(6):1921–3. doi: 10.1111/all.15290 (PMC9321988; doi:10.1111/all.15290)
Supplement: Supplementary file 1 — App S1 [file ALL-77-1921-s001.docx]

**online supplementary material**

**Tropomyosin is no accurate marker allergen for diagnosis of shrimp allergy in Central Europe**

João Grilo, MSc^1^, Ute Vollmann^1^, Martina Aumayr^2^, Gunter J. Sturm^3,4^, MD, PhD*, and Barbara Bohle, PhD^1^*

*contributed equally

^1^Department of Pathophysiology and Allergy Research, Medical University of Vienna, Vienna, Austria

^2^MacroArray Diagnostics, Vienna, Austria

^3^Allergy Outpatient Clinic Reumannplatz, Vienna, Austria
^4^Department of Dermatology and Venereology, Medical University of Graz, Graz, Austria

**Study cohort**

Serum samples were collected for routine allergy diagnosis from individuals presenting with shrimp-induced allergic reactions at the Allergy Outpatient Clinic Reumannplatz in Vienna and at the Department of Dermatology and Venereology, Medical University of Graz in Austria. Allergen-specific and total IgE levels were measured using ImmunoCAP 250 (Thermo Fisher Scientific, Waltham, USA). Allergen-specific IgE values >0.35 kU_A_/l to the shrimp mix containing *Pandalus borealis*, *Penaeus monodon*, *Metapenaeopsis barbata* and *Metapenauer joyneri* and to *Dermatophagoides pteronyssinus* were considered positive. Patients´ histories of their symptoms after consumption of shrimp were recorded and graduated into local and systemic allergic symptoms. The following symptoms were considered as local reactions: itchy mouth, scratchy throat, swelling of the lips, mouth, tongue, and throat. Systemic allergic reactions comprised generalized skin symptoms (urticaria, angioedema), dizziness, dyspnea, drop of blood pressure, and loss of consciousness. Experiments were performed according to the Declaration of Helsinki principles after approval by the ethics committee of the Medical University of Vienna (EK 1344/2018).

**Table S1.** Demographics of shrimp-allergic patients (n=79)

| **Characteristics** |  |
| --- | --- |
| **Sex (female/male), n (%)** | 34/45 (43/57) |
| **Age, median (range)** | 34 (14-79) years |
| **Local/systemic reactions to shrimp, n (%)** | 22/57 (28/72) |
| **Total serum IgE, median (25-75% percentile)** | 221 (108-450) kU/l |
| **shrimp-specific IgE, median (25-75% percentile)** | 2.6 (1.15-6.1) kU_A_/L |

**Allergy Explorer-ALEX2®**

The macroarray Allergy Explorer-ALEX2® (MacroArrayDx, Vienna, Austria) consists of a nitrocellulose membrane onto which nanoparticles activated with different allergens, allergen extracts and other components are spotted. Sera from shrimp-allergic and non-atopic subjects were diluted 1:5 in a diluent supplemented with an inhibitor of cross-reactive carbohydrate determinants (CCD) and incubated on the chips for 2 hours at RT. After washing, an alkaline phosphatase-labeled anti-human IgE antibody was added and incubated for 30 min at RT. Enzyme substrate was added and the intensity of the color reaction for each allergen spot was measured by a couple-charged device camera. The dedicated software digitalizes the images. Calibration is done *via* heterologous calibration against an IgE reference curve. Specific IgE values greater than 0.3 kU_A_/l were considered positive.

**Table S2.** Sensitization to shrimp and HDM allergens in ImmunoCAP and Allergy Explorer-ALEX2®

| **No.** | **Shrimp**  **CAP** | **Pen m 1** | **Pen m 2** | **Pen m 3** | **Pen m 4** | **Cra c 6** | **HDM**  **CAP** | **Der p 10** | **Der p 20** |
| --- | --- | --- | --- | --- | --- | --- | --- | --- | --- |
| **1** | 4.3 | 2.3 | 21.8 | <0.3 | 1.94 | <0.3 | 6.4 | 1.9 | 20.2 |
| **2** | 86.8 | 31.3 | <0.3 | 0.5 | 10.4 | 2.5 | 34.2 | 30.9 | <0.3 |
| **3** | 5.9 | 5.8 | 7.2 | <0.3 | <0.3 | <0.3 | 5.3 | 6.6 | 5.8 |
| **4** | 2.3 | <0.3 | <0.3 | <0.3 | 9.1 | <0.3 | 2.6 | <0.3 | <0.3 |
| **5** | 34.2 | 32.5 | 6.8 | <0.3 | 3.6 | 4.0 | 12.6 | 28.5 | 8.4 |
| **6** | 6.9 | <0.3 | 14.6 | <0.3 | 1.2 | <0.3 | 0.6 | <0.3 | 10.5 |
| **7** | 0.9 | 3.4 | <0.3 | <0.3 | <0.3 | <0.3 | <0.35 | 2.1 | <0.3 |
| **8** | 4.0 | <0.3 | 7.2 | <0.3 | 8.4 | <0.3 | 6.8 | <0.3 | 9.8 |
| **9** | 1.6 | 3.1 | 10.7 | <0.3 | <0.3 | <0.3 | 1.0 | 5.3 | 2.3 |
| **10** | 3.9 | <0.3 | <0.3 | <0.3 | <0.3 | <0.3 | 4.8 | <0.3 | <0.3 |
| **11** | 2.5 | <0.3 | <0.3 | 1.4 | <0.3 | <0.3 | 57.2 | <0.3 | <0.3 |
| **12** | 9.3 | <0.3 | <0.3 | <0.3 | 23.8 | <0.3 | <0.35 | <0.3 | <0.3 |
| **13** | 1.1 | 1.6 | <0.3 | 1.6 | <0.3 | <0.3 | 11.1 | 0.7 | <0.3 |
| **14** | 100 | 29.9 | 10.9 | <0.3 | <0.3 | <0.3 | 20.2 | 29.9 | 20.7 |
| **15** | 3.4 | 5.5 | <0.3 | <0.3 | <0.3 | <0.3 | 1.3 | 4.8 | <0.3 |
| **16** | 58.7 | 31.7 | 10.9 | <0.3 | 7.8 | 0.8 | n.t. | 30.2 | 4.1 |
| **17** | 2.9 | <0.3 | <0.3 | <0.3 | 9.1 | <0.3 | <0.35 | <0.3 | <0.3 |
| **18** | 2.5 | <0.3 | <0.3 | <0.3 | 7.0 | <0.3 | <0.35 | <0.3 | <0.3 |
| **19** | 0.4 | <0.3 | <0.3 | <0.3 | <0.3 | <0.3 | <0.35 | <0.3 | <0.3 |
| **20** | 6.3 | 9.6 | <0.3 | <0.3 | <0.3 | <0.3 | 4.4 | 17.3 | <0.3 |
| **21** | 0.5 | <0.3 | <0.3 | <0.3 | <0.3 | <0.3 | 2.6 | <0.3 | <0.3 |
| **22** | 0.6 | 1.4 | <0.3 | <0.3 | <0.3 | <0.3 | <0.35 | 1.2 | <0.3 |
| **23** | 0.9 | <0.3 | <0.3 | <0.3 | 4.6 | <0.3 | <0.35 | <0.3 | <0.3 |
| **24** | 5.8 | <0.3 | <0.3 | <0.3 | 18.4 | <0.3 | n.t. | <0.3 | <0.3 |
| **25** | 2.7 | <0.3 | <0.3 | <0.3 | <0.3 | <0.3 | 2.1 | <0.3 | <0.3 |
| **26** | 0.7 | 2.9 | <0.3 | <0.3 | <0.3 | <0.3 | <0.35 | 1.7 | <0.3 |
| **27** | 2.0 | <0.3 | <0.3 | <0.3 | 6.1 | 1.5 | <0.35 | <0.3 | <0.3 |
| **28** | 4.5 | 7.1 | 0.6 | <0.3 | <0.3 | <0.3 | 20.5 | 6.6 | 1.8 |
| **29** | 3.7 | <0.3 | <0.3 | <0.3 | 10.6 | <0.3 | <0.35 | <0.3 | <0.3 |
| **30** | 1.9 | <0.3 | <0.3 | <0.3 | <0.3 | <0.3 | 1.6 | <0.3 | <0.3 |
| **31** | 17.3 | 22.9 | 1.1 | <0.3 | <0.3 | <0.3 | 2.8 | 19.2 | 8.2 |
| **32** | 1.4 | <0.3 | <0.3 | <0.3 | <0.3 | <0.3 | <0.35 | <0.3 | <0.3 |
| **33** | 0.9 | <0.3 | <0.3 | <0.3 | <0.3 | <0.3 | 1.1 | <0.3 | <0.3 |
| **34** | 1.0 | 4.6 | <0.3 | <0.3 | <0.3 | <0.3 | 63.2 | 2.3 | <0.3 |
| **35** | 1.4 | <0.3 | <0.3 | <0.3 | 6.7 | <0.3 | <0.35 | <0.3 | <0.3 |
| **36** | 2.6 | 7.4 | <0.3 | <0.3 | <0.3 | <0.3 | 0.8 | 6.9 | <0.3 |
| **37** | 0.7 | <0.3 | <0.3 | <0.3 | 4.1 | <0.3 | <0.35 | <0.3 | <0.3 |
| **38** | 0.5 | <0.3 | 0.2 | 1.4 | <0.3 | <0.3 | 5.5 | <0.3 | <0.3 |
| **39** | 2.1 | <0.3 | <0.3 | <0.3 | <0.3 | <0.3 | n.t. | <0.3 | <0.3 |
| **40** | 0.9 | 3.1 | <0.3 | <0.3 | <0.3 | <0.3 | 1.6 | 4.4 | <0.3 |
| **41** | 3.1 | 6.3 | <0.3 | <0.3 | 0.2 | <0.3 | 2.3 | 4.5 | <0.3 |
| **42** | 4.1 | <0.3 | <0.3 | <0.3 | <0.3 | <0.3 | <0.35 | <0.3 | <0.3 |
| **43** | 0.9 | <0.3 | <0.3 | <0.3 | <0.3 | <0.3 | 3.7 | <0.3 | <0.3 |
| **44** | 0.6 | <0.3 | <0.3 | <0.3 | <0.3 | <0.3 | 3.9 | <0.3 | <0.3 |
| **45** | 1.6 | 0.4 | <0.3 | 1.6 | <0.3 | <0.3 | n.t. | <0.3 | <0.3 |
| **46** | 54.6 | 16.8 | 24.4 | 19.1 | 11.2 | 8.3 | 5.0 | 11.4 | 13.6 |
| **47** | 1.2 | <0.3 | <0.3 | <0.3 | <0.3 | <0.3 | 6.7 | <0.3 | <0.3 |
| **48** | 24.7 | 26.54 | <0.3 | <0.3 | <0.3 | <0.3 | 8.8 | 27.2 | <0.3 |
| **49** | 23.9 | 27.0 | 0.29 | <0.3 | <0.3 | <0.3 | 14.7 | 25.1 | 0.4 |
| **50** | 0.9 | <0.3 | <0.3 | <0.3 | <0.3 | <0.3 | 0.8 | <0.3 | <0.3 |
| **51** | 11.0 | 16.9 | 5.4 | <0.3 | <0.3 | <0.3 | 5.3 | 13.2 | 5.0 |
| **52** | 20.6 | 27.1 | <0.3 | <0.3 | <0.3 | <0.3 | 5.5 | 25.0 | <0.3 |
| **53** | 1.9 | 1.8 | <0.3 | <0.3 | <0.3 | <0.3 | 3.1 | 1.2 | <0.3 |
| **54** | 4.0 | <0.3 | <0.3 | <0.3 | <0.3 | <0.3 | 9.0 | <0.3 | <0.3 |
| **55** | 0.5 | <0.3 | <0.3 | <0.3 | <0.3 | <0.3 | 0.5 | <0.3 | <0.3 |
| **56** | 0.7 | <0.3 | <0.3 | <0.3 | <0.3 | <0.3 | 0.8 | <0.3 | <0.3 |
| **57** | 0.7 | <0.3 | <0.3 | <0.3 | <0.3 | <0.3 | n.t. | <0.3 | <0.3 |
| **58** | 2.7 | <0.3 | <0.3 | <0.3 | <0.3 | <0.3 | 3.4 | <0.3 | <0.3 |
| **59** | 2.3 | <0.3 | 10.5 | <0.3 | <0.3 | <0.3 | 33.1 | <0.3 | 6.6 |
| **60** | 5.9 | <0.3 | <0.3 | <0.3 | 20.4 | <0.3 | n.t. | <0.3 | <0.3 |
| **61** | 5.7 | <0.3 | <0.3 | <0.3 | 22.0 | <0.3 | n.t. | <0.3 | <0.3 |
| **62** | 3.7 | 0.3 | <0.3 | <0.3 | <0.3 | 4.3 | 1.8 | 0.5 | <0.3 |
| **63** | 48.8 | <0.3 | <0.3 | <0.3 | <0.3 | <0.3 | 27.4 | <0.3 | <0.3 |
| **64** | 9.7 | 1.3 | <0.3 | <0.3 | <0.3 | <0.3 | 40.9 | <0.3 | <0.3 |
| **65** | 4.4 | <0.3 | <0.3 | <0.3 | <0.3 | <0.3 | 19.7 | <0.3 | <0.3 |
| **66** | 23.4 | <0.3 | <0.3 | <0.3 | <0.3 | <0.3 | 2.8 | <0.3 | <0.3 |
| **67** | 0.5 | 23.5 | <0.3 | <0.3 | <0.3 | <0.3 | <0.35 | 17.3 | <0.3 |
| **68** | 1.2 | <0.3 | 0.7 | <0.3 | <0.3 | <0.3 | 0.5 | <0.3 | 1.1 |
| **69** | 1.5 | <0.3 | <0.3 | <0.3 | <0.3 | <0.3 | 0.4 | <0.3 | <0.3 |
| **70** | 1.6 | <0.3 | 4.3 | <0.3 | <0.3 | 7.5 | 2.3 | <0.3 | 21.6 |
| **71** | 1. | 9.6 | <0.3 | <0.3 | <0.3 | <0.3 | 15.1 | 8.9 | <0.3 |
| **72** | 1.3 | <0.3 | <0.3 | <0.3 | <0.3 | <0.3 | 1.2 | <0.3 | <0.3 |
| **73** | 20.7 | <0.3 | <0.3 | <0.3 | 26.7 | <0.3 | <0.35 | <0.3 | <0.3 |
| **74** | 5.5 | 0.9 | <0.3 | <0.3 | 33.2 | <0.3 | 53.6 | 0.6 | <0.3 |
| **75** | 57.1 | <0.3 | <0.3 | 3.1 | 9.1 | <0.3 | <0.35 | <0.3 | <0.3 |
| **76** | 1.4 | <0.3 | <0.3 | <0.3 | 6.5 | <0.3 | <0.35 | <0.3 | <0.3 |
| **77** | 21.3 | 10.1 | 13.1 | 9.4 | 6.1 | 4.2 | 2.7 | 7.4 | 6.4 |
| **78** | 8.7 | 9.5 | <0.3 | <0.3 | <0.3 | 3.8 | 6.7 | 8.3 | <0.3 |
| **79** | 0.5 | <0.3 | <0.3 | <0.3 | <0.3 | <0.3 | 0.7 | <0.3 | <0.3 |

**Immunoblotting studies**

For IgE-profiling to natural shrimp allergens we employed *Litopenaeus vannamei* (*Lit v*, whiteleg shrimp) because this species is frequently purchased and consumed in Austria and was therefore expected to be the cause of most allergic reactions in our study cohort. Furthermore, allergens described in *Pen m* have also been identified in *Lit v* (1-3). Four frozen, blanched, and beheaded (absent cephalothorax) whiteleg shrimps with a total weight of approximately 58 g were deveined and their tail shells were removed. Four tablets of EDTA-free cOmplete™ protease inhibitor cocktail (F. Hoffmann-La Roche AG, Basel, Switzerland) were dissolved in 200 ml of PBS at 4 ºC. Shrimps were added and homogenized in an electrical blender. The suspension was centrifuged at 15.000 x g for 20 min at 4 ºC. The supernatant was collected and dialyzed against PBS at 4 ºC using a 3.5 kDa cutoff membrane. The protein concentration was determined by BCA™ Protein Assay Kit (Thermo Fisher Pierce, Rockford, IL, USA). The protein extract was aliquoted and stored at -20 ºC until further use.

The *Lit v* protein extract (330 µg) was separated after cooking at 95°C for 3 minutes by 15% SDS-PAGE under reducing conditions and electroblotted onto a nitrocellulose membrane (Cytiva, Malborough, MA, USA) for 60 minutes at 4 °C. Nitrocellulose membrane strips (0.5 cm) were cut and saturated with gold buffer, which is PBS supplemented with 0.5%Tween 20, 0.5% BSA, and 0.05% NaN_3_ for 30 min at RT. Then strips were incubated with the individual sera from shrimp-allergic and non-atopic subjects (diluted 1:4 in gold buffer) overnight at 4 ºC. After washing, bound IgE was detected with a ^125^I-labelled anti-human IgE antibody (Demeditec Diagnostics, Kiel, Germany, diluted 1:10 in gold buffer) incubated overnight at 4 ºC. After washing, strips were left do dry at room temperature and exposed to X-ray films at -80 ºC for 3 to 5 days.

**References**

1. Ayuso R, Grishina G, Bardina L, Carrillo T, Blanco C, Ibanez MD, et al. Myosin light chain is a novel shrimp allergen, Lit v 3. J Allergy Clin Immunol 2008;122(4):795-802.

2. Ayuso R, Grishina G, Ibanez MD, Blanco C, Carrillo T, Bencharitiwong R, et al. Sarcoplasmic calcium-binding protein is an EF-hand-type protein identified as a new shrimp allergen. J Allergy Clin Immunol 2009;124(1):114-120.

3. Garcia-Orozco KD, Aispuro-Hernandez E, Yepiz-Plascencia G, Calderon-de-la-Barca AM, Sotelo-Mundo RR. Molecular characterization of arginine kinase, an allergen from the shrimp Litopenaeus vannamei. Int Arch Allergy Immunol 2007;144(1):23-28.
